# Cell death-based treatment of various diseases: a fifty-year journey

**DOI:** 10.1038/s41419-017-0168-3

**Published:** 2018-01-25

**Authors:** Vitaliy O. Kaminskyy, Boris Zhivotovsky

**Affiliations:** 1 Institute of Environmental Medicine, Karolinska Institutet, Box 210, 171 77 Stockholm, Sweden; 2 Faculty of Medicine, Lomonosov Moscow State University, Moscow, Russia 119991

## Body

More than 50 years ago, Anna Kane Laird suggested that the retardation of tumor growth observed by researchers and clinicians might be due to an active decrease in the tumor growth rate or to cell death. She postulated that “stimulation of natural growth-retarding factors might be of practical importance in reducing the incidence of clinical cancer, i.e., the systemic illness and death of the host, if tumor growth could be retarded enough to bring it to an upper limit that is within the physiological tolerance of the host”^1^. Based on mathematical analysis a subsequent study stated that death of normal or neoplastic cells could be a predestined, genetically determined phenomenon^2^. In 1972, Kerr, Wyllie, and Currie coined the term “apoptosis” to describe a basic biological phenomenon with wide-ranging implications in tissue kinetics^3^. They postulated that apoptosis can occur spontaneously in untreated malignant neoplasms, and is involved in therapeutically induced tumor regression. To date, more than ten different cell death modalities have been recognized by the Nomenclature Committee on Cell Death^4^. Disturbances in cell death pathways at the molecular level can be linked to the pathogenesis not only of cancer, but also other diseases of enormous social importance, such as HIV, atherosclerosis, ischemia, reperfusion injury, infection, inflammation, autoimmune, and neurological disorders^5^. An accumulation of data testifies the existence of cross talk between the various cell death mechanisms. There is also considerable evidence that suppression or activation of one type of cell death can influence the activity of another and that the balance between them can affect the response of cells to treatment. Nowadays there is evidence that tissue-specific and organ-specific activation of cell death mechanisms influences how various diseases respond to treatment. Thus, the therapies for kidney-associated diseases are linked to efficient necroptosis or ferroptosis, therapies for neutrophil-associated disorders are linked to necroptosis etc. Tiny perturbations in the regulation of cell death pathways could be potential targets for improvements of therapy. With this in mind, many research groups and pharmaceutical companies are focusing on development of new compounds that act against specific diseases by targeting cell death pathways. In the last decade FDA approved many drugs targeting cell death processes for treatment of various diseases. Amongst these are anti-cancer drugs. In 2016, the FDA approved a highly selective small-molecule Bcl-2 inhibitor, venetoclax (ABT-199) for the oral treatment of AML and CLL. A selective inhibitor of Bcl-W, navitoclax (ABT-263), has been proposed as a treatment for advanced and recurrent small-cell lung carcinomas. An antibody against programmed cell death protein 1 (anti-PDL1) was approved for treatment of bladder cancer and nivolumab, which also targets PD-1, for non-small-cell lung cancer, melanoma, and renal cell cancer. A number of p53-Mdm2 inhibitors, including Nutlin and MI-773, as well as reactivators of mutant p53, including APR-246 and SCH529074, are at different stages of clinical trials involving various cancers.

Interestingly, the combination of a novel caspase inhibitor IDN-7314 with 5-fluorouracil synergistically blocked tumor growth compared with 5-fluorouracil alone, via induction of necroptosis^6^. However, activation of necroptosis does not always imply successful therapy. Necroptosis is involved in the regulation of pathogenesis of inflammatory diseases, suggesting that triggering necroptosis in cases of Crohn’s disease or inflammatory skin disease might have off-target effects, leading to pathological inflammation of normal tissues. These concerns should be taken into consideration during the planning of treatment. With these concerns in mind, Infliximab (Remicade) was successfully tested as a potential treatment for Crohn’s disease, which is associated with inflammasome dysfunction.

Recent achievements in development of cell death-based approaches to treatment of various diseases are summarized in a series of eleven review articles published in this issue of Cell Death & Disease.

The role of necroptosis in neutrophil cell death is discussed by Wang and colleagues^7^. The authors highlight the key inducers of necroptosis in neutrophils. Thus, inhibition of caspase-8 activates apoptosis in response to the presence of the TNF ligand and RIPK3-dependent necroptosis can be triggered by phagocytosis of *S. aureus*. However, under some conditions, TNFR1 stimulation via TNF triggers other cellular pathways, such as activation of NF-kB signaling or apoptotic cell death. Since neutrophils are involved in various types of tissue inflammation and disease, it is important to understand the different mechanisms regulating neutrophil necroptosis in order to uncover novel drug targets for treatments of autoimmune and inflammatory diseases.

In addition to necroptosis there are another two forms of regulated necrosis, namely ferroptosis and pyroptosis, which have been intensively studied in the context of acute kidney injury (AKI) and transplantation. The immunological consequences of kidney cell death are discussed by Sarhan and colleagues^8^. By contributing to AKI, regulated necrosis causes antibody-mediated rejection. AKI promotes loss of the majority of tubular cells by ferroptosis, resulting in peroxidation of membrane lipids. Although elimination of cells by apoptosis is beneficial in many physiological conditions, the integrity of plasma membrane during apoptosis prevents it being immunogenic. It seems that pyroptosis is the most immunogenic pathway due to its role in maturation of IL-1 and IL-18 alongside the release of inflammatory caspases. However, production of IL-33 and CXCL1 during necroptosis results in inhibition of innate immunity.

Martin-Sanchez and colleagues^9^ describe the role of different cell death modalities in the pathogenesis and therapy of urinary tract-associated diseases. In cancer of urinary tract there are several key processes that lead to escape from apoptosis, including autophagy, downregulation of Fas and caspase-3 as well as upregulation of Bcl-2. A deficiency of VHL in renal cell carcinoma makes it resistant to NK cells and hypoxia-induced cell death, whilst inflammatory signaling induced by NF-kB promotes necroptosis. Tumors of the urinary tract develop resistance to cell death, but infection of urothelial cells with uropathogenic *E. coli* may, depending on the bacterial strain, prevent host cell death or promote cell death by apoptosis, necrosis or an iron-dependent mechanism.

It is known that diabetes is characterized by hyperglycemia and involves significant loss of insulin-producing pancreatic β cells via several cell death mechanisms, including apoptosis, necroptosis and pathological necrosis. Oliveira Volpe and colleagues describe the therapeutic targets for controlling this hyperglycemia-triggered metabolic pathway, which is one of the causes of diabetic complications, by down-regulating oxidative stress in β cells^10^. Hyperglycemia-mediated upregulation of ROS is associated with activation of several metabolic routes, including diacylglycerol—protein kinase C—and NADPH-oxidase pathway. The authors suggest that inhibitors of this pathway could be used to control ROS and prevent cell death in diabetes.

Christina Ising and Michael Henneka focus on the role of innate immune mechanisms in damage of neurons during neurodegeneration^11^. It has been shown that inflammatory mediators released by microglia and astrocytes contribute to the pathogenesis of neurodegenerative disease. Under healthy conditions microglial cells generate and secrete factors (e.g., BDNF) essential for neuronal survival but, after activation, they secrete various pro-inflammatory cytokines, such as IL-1β and immunomodulatory factors e.g., nitric oxide, which have a negative impact on neuronal function, structure and survival.

The role of the tumor vasculature in anti-tumor immunity and immunotherapy is a hot topic in cancer biology and is summarized by Schaaf and colleagues^12^. Activated CD8+ T cells can recognize tumor-associated antigens at the surface of tumor cells and promote tumor cell death via the perforin-granzyme and/or FasL/TRAIL systems. However, the tumor vasculature creates physical and functional barriers to infiltration of immune cells. The authors describe the mechanisms by which tumor-associated blood vasculature promotes an immunoresistant tumor microenvironment. At present, several methods of stimulating anti-tumor immunity with IL-2, TNF-α, and IFN-γ are subject to intensive investigation.

Amplification of MYCN and mutations in anaplastic lymphoma kinase (ALK) are considered the main drivers of neuroblastoma (NB). Valter and colleagues discuss various pathways that allow NB cells to escape death^13^. It has been suggested that MYCN plays a role in regulation and expression of p53 and Tap73 and their relation to apoptosis in NBs. Hypoxia promotes activation of several factors, including HIF-1 that can block apoptosis by inhibiting p53 or promote tumor growth by stimulation of glycolysis and angiogenesis. Hence, it has been proposed that inhibitors of HIF-1 could promote apoptosis in NB cells. Since many NB cells do not express caspase-8, treatments that increase expression of necroptotic proteins are amongst the promising strategies for triggering necroptosis in NBs.

Simone Fulda^14^ discusses potential routes to reactivation of pathways triggering cell death in glioblastomas. Current treatment regimens for glioblastoma include radiation and chemotherapy with the alkylating agent temozolomide (TMZ). It has been suggested that SMAC mimetics operate synergistically with TMZ by promoting apoptotic cell death. This mechanism would engage NF-κB-dependent upregulation of interferon-β, which promotes apoptosis by upregulating Bax and Puma. Other treatments for glioblastoma include targeting TRAIL death receptors in combination with kinase or HDAC inhibitors as well as BH3 mimetics. Thus, reactivation of apoptosis is one of the main aims of glioblastoma treatments.

Cell death-based therapy of melanoma is discussed by Mattia and colleagues^15^. Increased expression of the DNA repair protein O6-alkylguanine DNA alkyltransferase (MGMT) leads to melanomas resistance to alkylating agents. The necroptosis pathway is often deregulated in melanoma because of low RIPK3 expression. Targeted therapies for melanomas includes BRAF and MEK inhibitors. Immunotherapies with the antibody blocking CTLA-4 prevents immunosuppression of lymphocytes, and reactivation of effector T lymphocytes. Another approach to sensitization of tumors is to restore the functions of tumor-suppressive cell death pathways by applying epigenetic compounds.

Denisenko and colleagues discuss signals driving growth of lung adenocarcinoma and cell death pathways induced by various therapeutic approaches to this tumor type^16^. Lung adenocarcinoma is characterized by mutations in epidermal growth factor receptor (EGFR) and KRAS, fusion in the ALK oncogene and alterations in different kinase signaling cascades. The FDA has approved several tyrosine kinase inhibitors (TKI) for therapy of lung adenocarcinoma. However, resistance to TKI inhibitors may be developed via enhancement of antiapoptotic signals such as NF-kB. Therefore, several combinations of chemotherapy plus antibody-targeted therapies are now in clinical trials.

Cell death-based therapies of childhood cancer are discussed by Westhoff and colleagues^17^. These authors describe the ways in which malignant cells escape pharmacological inhibition of kinase, such as mutation or upregulation of tyrosine kinase receptors. Several kinase inhibitors have been suggested as potential treatments for pediatric tumors. These tumors often exhibit resistance to death-receptor targeted therapy due to downregulation of caspase-8. BH3-mimetics have been shown to be potent treatments in high-risk groups of patients with ALL and NBs, whereas Smac mimetics are to “sensitize” pediatric tumors to apoptosis. Thus, cell death-based treatments are one of the main forms of precision therapy for childhood cancers.

In the almost 50 years since cell death was first proposed as a potential target for cancer therapy is has become obvious that targeted cell death-based treatment has an important role to play in successful therapy of various human diseases. Various approaches to regulation of cell survival and cell death are currently in use. This issue of *Cell Death & Disease* contains reviews summarizing recent achievements in the field of cell death-based treatment of a number of diseases. We hope that this collection of articles will attract scientists’ attention to this growing area of research, which offers many opportunities to exploit cell death mechanisms to develop new treatments for human disease.
